# Development of a UPLC–MS/MS method for simultaneous therapeutic drug monitoring of anti-hepatocellular carcinoma drugs and analgesics in human plasma

**DOI:** 10.3389/fphar.2023.1136735

**Published:** 2023-05-31

**Authors:** Shijia Lu, Mingming Zhao, Limei Zhao, Guofei Li

**Affiliations:** Department of Pharmacy, Shengjing Hospital of China Medical University, Shenyang, China

**Keywords:** UPLC-MS/MS, hepatocellular carcinoma, chemotherapy and targeted drugs, analgesics, human plasma, therapeutic drug monitoring

## Abstract

In hepatocellular carcinoma treatment, sorafenib, oxaliplatin, 5-fluorouracil, capecitabine, lenvatinib, and donafenib are first-line drugs; regorafenib, apatinib, and cabozantinib are second-line drugs; and oxycodone, morphine, and fentanyl are commonly used analgesics. However, the high degree of inter- and intra-individual variability in the efficacy and toxicity of these drugs remains an urgent issue. Therapeutic drug monitoring (TDM) is the most reliable technical means for evaluating drug safety and efficacy. Therefore, we developed an ultra-performance liquid chromatography–tandem mass spectrometry (UPLC–MS/MS) method for simultaneous TDM of three chemotherapy drugs (5-fluorouracil, oxaliplatin, and capecitabin), six targeted drugs (sorafenib, donafenib, apatinib, cabozantinib, regorafenib, and lenvatinib), and three analgesics (morphine, fentanyl, and oxycodone). We extracted 12 analytes and isotope internal standards (ISs) from plasma samples by magnetic solid phase extraction (mSPE) and separated them using a ZORBAX Eclipse Plus C18 column with water containing 0.1% formic acid and methanol containing 0.1% formic acid as the mobile phase. The analytical performance of our method in terms of sensitivity, linearity, specificity, carryover, precision, limit of quantification, matrix effect, accuracy, dilution integrity, extraction recovery, stability, and crosstalk of all the analytes under different conditions met all the criteria stipulated by the guidelines of the Chinese Pharmacopoeia and U.S. Food and Drug Administration. The response function was estimated at 10.0–10 000.0 ng/mL for sorafenib, donafenib, apatinib, cabozantinib, regorafenib, and lenvatinib, and 20.0–20 000.0 ng/mL for 5-fluorouracil, oxaliplatin, capecitabin, morphine, fentanyl, and oxycodone, with a correlation of > 0.9956 for all compounds. The precision and accuracy of all analytes were < 7.21% and 5.62%, respectively. Our study provides empirical support for a simple, reliable, specific, and suitable technique for clinical TDM and pharmacokinetics.

## 1 Introduction

Hepatocellular carcinoma (HCC) is a common primary liver cancer with high mortality and high rates of morbidity, especially in Asia and Africa. (Piñero et al., 2020; Cucarull et al., 2022). China has a high incidence of liver cancer, with new cases and deaths exceeding 50% of the global total value (Liu and Song, 2021). Although a small number of patients are effectively diagnosed and treated in the early stage of HCC, the recurrence rate remains high. The current treatment of HCC is mainly based on traditional chemo- and targeted drug therapy, though, for the vast majority of patients with primary HCC, there is no effective clinical treatment, and the prognosis is poor.

According to the Chinese Society of Clinical Oncology (CSCO) guiding principles for the diagnosis and treatment of HCC, first-line drugs mainly include sorafenib, oxaliplatin, 5-fluorouracil, capecitabine, lenvatinib, and donafenib, and second-line drugs include regorafenib, apatinib, and cabozantinib (Fan et al., 2022). Effective relief from cancer pain is an essential complementary requirement of effective treatment, for which morphine, oxycodone, and fentanyl are commonly used clinically effective analgesics (Bardwell et al., 2019; Zhou et al., 2020; Schmidt-Hansen et al., 2022). Although these drugs play an important role in inhibiting the progression of liver cancer, they show considerable individual variation in efficacy and toxicity (Man et al., 2021). In general, plasma drug concentrations are positively correlated with drug efficacy and toxicity, which means that appropriate drug concentrations are essential to treatment success. Low plasma drug concentrations cannot exert anti-tumor and analgesic effects, leading to tumor progression or recurrence, pain, and even mania in some patients, whereas high plasma drug concentrations can cause side effects, including myelosuppression, hypertension, and hypoventilation, among others. Several factors can affect drug plasma concentration, including genetic polymorphism of metabolic enzymes or transporters, drug–drug interactions based on metabolic enzymes or transporters, development of multidrug resistance, decreased compliance, and changes in liver and kidney function, among others.

Certain techniques have been developed to monitor and maintain the plasma concentration of analgesic and anti-tumor drugs within the treatment window. Therapeutic drug monitoring (TDM) is the most commonly used technology to measure drug concentrations in individualized treatments. It has been applied for a variety of drugs, including antiepileptic, immunosuppressive, antidepressant, antitumor, antibacterial, and antipsychotic drugs. The method used for regular TDM mainly include UV spectrophotometry, LC–tandem mass spectrometry (LC–MS/MS), immunoassay and high-performance liquid chromatography (HPLC) (Kozo et al., 2017; Sommerfeld-Klatta et al., 2020; Tuzimski and Petruczynik, 2020). Among these, immunoassay and chromatography are the most commonly used. The immunoassay is time-efficient, and the sample processing method is simple, but it is susceptible to interference from endogenous substances and metabolites, and its specificity requires improvement. More importantly, the immunoassay method cannot determine drug concentrations simultaneously. In contrast, the LC–MS/MS technology effectively addresses the deficiencies of the immunoassay method (Li et al., 2021; Llopis et al., 2021; Qi and Liu, 2021). LC–MS/MS can be used to monitor dozens of drugs simultaneously using the MRM mode. It also shows good specificity and is almost unaffected by endogenous substances of plasma under optimized conditions. Therefore, TDM using LC–MS/MS has seen considerable clinical application and development.

The goal of our paper was to establish a sensitive UPLC–MS/MS method for the simultaneous determination of three chemotherapy drugs [oxaliplatin (OXA), 5-fluorouracil (5-Fu), and capecitabine (CAP)], six targeted drugs [sorafenib (SOR), lenvatinib (LEN), donafenib (DON), regorafenib (REG), apatinib [APA], and cabozantinib (CAB)], and three analgesics [morphine (MOR), oxycodone (OXY), and fentanyl (FEN)] in human plasma and study its feasibility in clinical TDM. The UPLC–MS/MS method allows for the quantification of plasma concentrations for several drugs across many patients, irrespective of individual prescriptions or treatment and thus saves a lot of costs and manpower. Additionally, the turnover for TDM results can be greatly improved by using the standard curve and quality control samples containing 12 analytes simultaneously, which would allow doctors to adjust dosing regimens in real-time. Isotopic-labeled compounds were chosen as internal standard (IS). Magnetic solid phase extraction (mSPE) method, novel SPE technique, was used to extract 12 analytes and ISs from plasma samples. The final results demonstrated that the newly developed method could simultaneously quantify the concentrations of 5-Fu, OXA, CAP, SOR, LEN, DON, REG, APA, CAB, MOR, OXY, and FEN in human plasma. The findings of our study could aid in the research of rational drug prescription and high-throughput TDM.

## 2 Materials and methods

### 2.1 Experimental reagents

**FIGURE 1:**
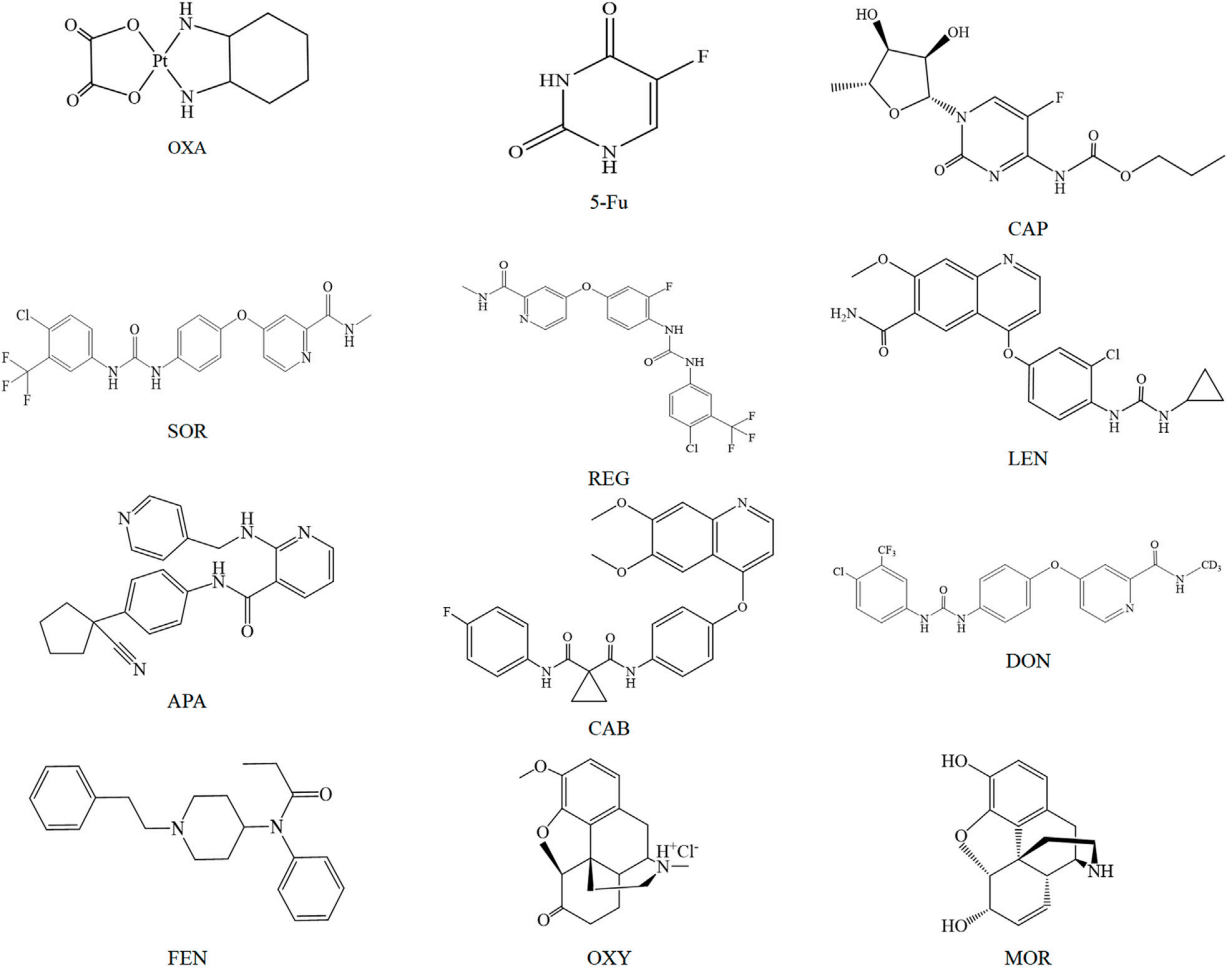
Chemical structure diagram of OXA, 5-Fu, CAP, SOR, REG, LEN, APA, CAB, DON, FEN, OXY, and MOR.

5-Fu, OXA, CAP, CAP-D11 and 5-Fu-^13^C were purchased from Sigma Aldrich (St. Louis, MO, United States). SOR, LEN, DON, REG, APA, CAB, LEN-D4, CAB-D4, SOR-^13^CD3 and REG-D3 were purchased from Toronto Research Chemicals (Ontario, Canada). MOR, OXY, FEN and MOR-D3 were provided by Sapphire Bioscience (Beaconsfifield, NSW, Australia). Acetonitrile, methanol and formic acid were purchased from Anaqur Chemicals (Wilmington, United States). A Milli-Q system was chosen as water purification system to prepare deionized water (Millipore, Milford, MA, United States). The structures of 5-Fu, OXA, CAP, SOR, REG, LEN, APA, DON, CAB, MOR, OXY, and FEN are illustrated in Figure 1.

### 2.2 Equipment and conditions

An Agilent 1290 UPLC system (Agilent, Santa Clara, CA, United States) was composed of two pumps for gradient flow (Model: G4220A) and was equipped with a column oven (model: G1316C), temperature controlled 54-well plate auto-sampler (model: G4226A) and in-line degasser (model: G-1330B). Chromatographic separation of analytes and ISs was performed by injecting plasma sample (2 μL) onto a reversed phase column (ZORBAX Eclipse Plus C18, 50.0 × 2.1 mm, 1.7 μm) at 30°C with a total run time of 3.5 min. Ultra pure water containing 0.1% formic acid and HPLC-grade methanol containing 0.1% formic acid were chosen as mobile phase, and the flow rate was set to 0.3 mL/min.

The MS spectrometric detection of the analytes and ISs was performed on a QTRAP 4,500 MS system (AB SCIEX). In the mass spectrometer, 12 analytes and 7 ISs were ionized under positive mode by an electrospray ionization (ESI) source with the optimal parameters: 4,500 V for ionizing voltage, 400°C for source temperature, 45 psi for GS1, 40 psi for GS2, 40 psi for curtain gas, 15 V for entrance potential and 20 V for collision exit potential. The compounds-dependent MS parameters of MRM channels, including collision energy (CE) and declustering potential (DP) were optimized independently in order to get a stable and strong signal and are shown in Table 1. Data acquisition and processing were performed by analyst software v1.6.2 (AB Sciex).

**TABLE 1:** Calibration range, calibration curves, coefficient of correlation (r) and MS parameters of analytes and ISs.

Drug	Calibration range (ng/mL)	Calibration curves	*r*	Transition (m/z)	Collision energy (V)	Declustering potential (s)
SOR	10.0–10,000.0	y = 1.8943x + 0.8392	0.9991	465.0→252.1	37	74
OXA	20.0–20,000.0	y = 0.7466x + 0.3563	0.9993	398.1→306.0	29	53
5-FU	20.0–20,000.0	y = 1.4872x + 1.0352	0.9993	128.9→41.9	28	50
CAP	20.0–20,000.0	y = 3.7118x + 1.7,659	0.9997	360.3→243.8	30	45
LEN	10.0–10,000.0	y = 0.8223x + 0.5910	0.9981	427.4→370.4	39	64
DON	10.0–10,000.0	y = 2.6396x + 1.4,820	0.9996	468.2→273.2	40	45
REG	10.0–10,000.0	y = 3.8836x + 1.7,641	0.9997	481.0→259.8	25	42
APA	10.0–10,000.0	y = 2.0043x + 0.9175	0.9984	398.1→212.0	30	45
CAB	10.0–10,000.0	y = 1.3572x + 0.6837	0.9992	502.0→323.0	42	51
MOR	20.0–20,000.0	y = 1.4738x + 0.6681	0.9992	286.2→152.1	41	57
FEN	20.0–20,000.0	y = 1.7403x + 0.6893	0.9989	337.3→187.9	36	50
OXY	20.0–20,000.0	y = 2.7317x + 0.8989	0.9982	316.3→297.9	38	40
SOR-^13^CD3	____	____	____	469.0→256.1	35	73
CAB-D4	____	____	____	506.0→323.0	38	53
CAP-D11	____	____	____	371.1→255.1	43	47
5-Fu-^13^C	____	____	____	131.9→43.9	35	40
REG-D3	____	____	____	484.5→263.1	45	38
LEN-D4	____	____	____	431.4→370.4	42	62
MOR-D3	____	____	____	289.2→152.0	40	55

### 2.3 Stock solutions, calibration curve, and quality control samples

Individual stock solutions of 5-Fu, OXA, CAP, SOR, LEN, DON, REG, APA, CAB, MOR, OXY, FEN, and CAP-D11 and 5-Fu-^13^C, LEN-D4, CAB-D4, SOR-^13^CD3 and REG-D3, MOR-D3 were prepared with methanol at a concentration of 500.0 μg/mL. 15% methanol was chosen as diluting solvent to dilute the stock solution to obtain series of concentration standard solutions.

Standard curves were plotted by plotting the peak area ratio of ISs against the corresponding analyte concentration. The linearity of the study was evaluated by evaluating three standard curves on three consecutive days. A set of mixed standard curves was obtained by adding 20 μL of mixed working solutions to 100 μL blank human plasma to obtain a series of concentrations: 10.0, 25.0, 50.0, 100.0, 250.0, 1,000.0, 5,000.0, and 10,000.0 *n*g/mL for DON, SOR, CAB, REG, APA, and LEN and 20.0, 50.0, 100.0, 200.0, 500.0, 2,000.0, 10,000.0, and 20,000.0 *n*g/mL for 5-Fu, OXA, CAP, MOR, OXY, and FEN.

The Quality control (QC) samples used to assess the accuracy of the method were prepared at four different level: lower limit of quantification (LLOQ), low QC (LQC, 2-fold of the LLOQ), medium QC (MQC, middle of calibration curve range) and high QC (HQC, 80% of upper limit of quantification). Similarly, the QC samples were also prepared by adding 20 μL of mixed working solutions to 100 μL blank human plasma, and the specific concentrations were: 10.0, 20.0, 200.0, 2,000.0 ,and 8,000.0 *n*g/mL for DON, SOR, CAB, REG, APA, and LEN and 20.0, 40.0, 400.0, 4,000.0, and 16,000.0 *n*g/mL for 5-Fu, OXA, CAP, MOR, OXY, and FEN. The LLOQ was 10.0 ng/mL for DON, SOR, CAB, REG, APA, and LEN and 20.0 ng/mL for 5-Fu, OXA, CAP, MOR, OXY, and FEN. By diluting the stock solution with 15% methanol, we obtained 100.0 *n*g/mL CAP-D11, 5-Fu-^13^C, and MOR-D3 solution and 200.0 *n*g/mL LEN-D4, CAB-D4, SOR-^13^CD3, and REG-D3 solution. Here, 5-Fu-^13^C was used as a common IS for 5-Fu, OXA and CAP; LEN-D4 was used as a common IS for APA and LEN; SOR-^13^CD3 was used as a common IS for DON and SOR; MOR-D3 was used as a common IS for MOR, OXY, and FEN; CAB-D4 and REG-D3 were used as IS of CAB and REG respectively.

### 2.4 Samples extraction

1) The analytes were extracted using a mSPE technique. The core of mSPE method was the mSPE particle, which consisted of a magnetically inert core and porous polymers pyrrolidone–divinylbenzene–polystyrene in the outer layer. The mSPE particles had the following characteristics: 1) Its surface had a large number of pores and a small particle size (about 30 μm), which made it have a great specific surface area and adsorption capacity, and thus can effectively separate small molecular weight analytes and high molecular weight endogenous substances, greatly improving the cleanliness of the sample. 2) The interaction between the external magnetic bar and mSPE particles can be easily used to wash the mSPE particles adsorbing drugs, which can effectively avoid the blockage of traditional SPE columns. The specific operation process was as follows: 1) The first step was to activate mSPE particles with methanol while removing surface impurities. Here, 30 μL of magnetic particleswith a concentration of 0.1 g/mL and 300 μL of activated solvent methanol were simultaneously added to the first column of sample plate, and continuously stirred for 1.5 min. 2) The second step was to use 500 μL of water to wash methanol off the surface of mSPE particles so as not to affect the subsequent drug adsorption. 3) The third step was to complete the adsorption of mSPE particles to 7 ISs and 12 analytes. We added 100 μL of blank human plasma, 20 μL of working solution and 20 μL of ISs solution (For clinical plasma samples, we added 100 μL of plasma sample, 20 μL of methanol and 20 μL of ISs solution) to the third column and continuously stirred for 1.5 min. 4) Similar to the second step, the fourth step also used ultra-pure water washing to remove impurities from the surface of the mSPE particles that adsorbed the drugs. The drug-adsorbed particles were adsorbed by magnetic bars and transferred to the next column, and were rinsed by 400 μL water to wash off methanol and endogenous substances such as salts, amines, fatty acids, glycerates, phospholipids, etc. 5) The final step was to elute the adsorbed 7 ISs and 12 analytes from mSPE particles for UPLC-MS/MS analysis. The drug-adsorbed particles were adsorbed by magnetic bars and transferred to the fifth column, and were rinsed by 500 μL acetonitrile for 1.5 min to wash off 7 ISs and 12 analytes. The magnetic bar did not directly contact the magnetic particles, but indirectly controled the magnetic particles through a sleeve during the whole mSPE process. The magnetic bar can move up and down and left and right freely. Therefore, the adsorption and desorption of magnetic particles can be realized by controlling the rise and fall of the magnetic bar.

### 2.5 Method validation

(National Pharmacopoeia Commission., 2020; U.S. Food and Drug Administration., 2018).

#### 2.5.1 Specificity

The specificity test was conducted by comparing chromatograms of eight different endogenous sources of blank human plasma, ISs, and clinical plasma samples (5-Fu, OXA, CAP, SOR, DON, CAB, REG, LEN, APA, MOR, OXY, and FEN). In addition, the specificity was also evaluated by monitoring 14 common drug combinations during tumor treatment, including 2 antiviral drugs (entecavir and tenofovir fumarate), seven antiemetics (aprepitant, ondansetron, dolastron, granisetron, palonosetron, methylprednisolone and dexamethasone), and five sedative drugs (midazolam, diphenhydramine, diazepam, lorazepam, andphenobarbital). The peak response of the endogenous components for every analyte should be *<* 20% and *<* 5% of the peak area of the LLOQ and the average peak area of the ISs respectively.

#### 2.5.2 Linearity, carryover, and LLOQ

Six-point standard curves (10.0–10 000.0 *n*g/mL for SOR, DON, REG, CAB, APA, and LEN and 20.0–20 000.0 *n*g/mL for 5-Fu, OXA, CAP, MOR, OXY, and FEN) in triplicate were determined and evaluated on 3 days. Meanshile, the linearity for SOR, DON, REG, CAB, APA, LEN, 5-Fu, OXA, CAP, MOR, OXY and FEN were assessed based on the weighted (1/x^2^) least squares linear regression of peak area ratio of IS against analyte concentration. The LLOQ and ULOQ were separately defined as the lowest concentration and the highest concentration of the standard curves. The impact of carryover was also evaluated using blank human samples after the analysis of ULOQ and should be < 20% of the LLOQ.

#### 2.5.3 Accuracy and precision

The inter-day precision and accuracy were studied by determining five replicates of HQC, MQC, LQC and LLOQ level on three consecutive days. The intra-day accuracy and precision were conducted at HQC, MQC, LQC and LLOQ level respectively by five replicates on a single assay. The relative standard deviation (RSD, %) and relative error (RE, %) were used to evaluate the accuracy and precision of the method. The results of precision and accuracy should be within ± 15%, while the criterion was within ± 20% for LLOQ.

#### 2.5.4 Recovery and matrix effect

Extraction recovery and the matrix effect for SOR, DON, CAB, REG, LEN, APA, 5-Fu, OXA, CAP, MOR, OXY, and FEN were assessed in the QCs and LLOQ concentrations. Extraction recovery was envestigated through comparing peak area ratio of extracted plasma samples to post-extraction plasma samples containing the same amount of the analytes (n = 6). The matrix effect was envestigated through comparing peak areas ratio of analytes added into post-extracted blank human plasma samples with 12 analytes diluted in pure water at corresponding concentrations(n = 6). As required, the results of the recoveries and matrix effects should be between 85% and 115%.

#### 2.5.5 Stability

The short-term stability (12 h/room temperature), three freeze-thaw stability (−20.0°C to room temperature) and long-term storage stability (30 days/−70°C) of SOR, DON, CAB, REG, LEN, APA, 5-Fu, OXA, CAP, MOR, OXY, and FEN in blank human plasma were studied by analyzing QC samples.

#### 2.5.6 Dilution integrity

The dilution of the plasma sample should not affect the precision and accuracy. Therefore, our study evaluated the dilution integrity of the method. A simulated plasma sample with concentration of 4 fold ULOQ and 40 fold ULOQ was prepared respectively by adding the analytes to the blank plasma. Then, the above simulated plasma samples were diluted to HQC level with blank plasma, and the dilution ratio was 5 and 50 fold respectively. As requirde, the RE and RSD values should be ≤ ± 15%.

### 2.6 Application

Steady-state trough concentration of SOR, DON, CAB, REG, LEN, APA, 5-Fu, OXA, CAP, MOR, OXY, and FEN in human plasma was selected as the TDM index. Clinical samples were acquireed from patients taking SOR, DON, CAB, REG, LEN, APA, 5-Fu, OXA, CAP, MOR, OXY, and FEN between July 2021 and May 2022. All patients agreed to use their non-genetic and clinical data for clinical research by signing consent. Clinical plasma samples and blank plasma samples came from the Laboratory and Hematology Department of Shengjing Hospital respectively. The Internal Review Boards were notified about this TDM project, but official approval was not required because no additional samples were needed. In addition, the procedures of the whole experiment were in accordance with the principles stated in the Declaration of Helsinki. The dosing schedule and blood collection time of each enrolled patient should be as similar as possible to avoid affecting the reliability of the results. Blood sample was centrifuged at 4,500 rpm for 6 min, and drugs were extracted from the obtained plasma using the mSPE technique.

## 3 Results and discussion

### 3.1 Method optimization

**FIGURE 2:**
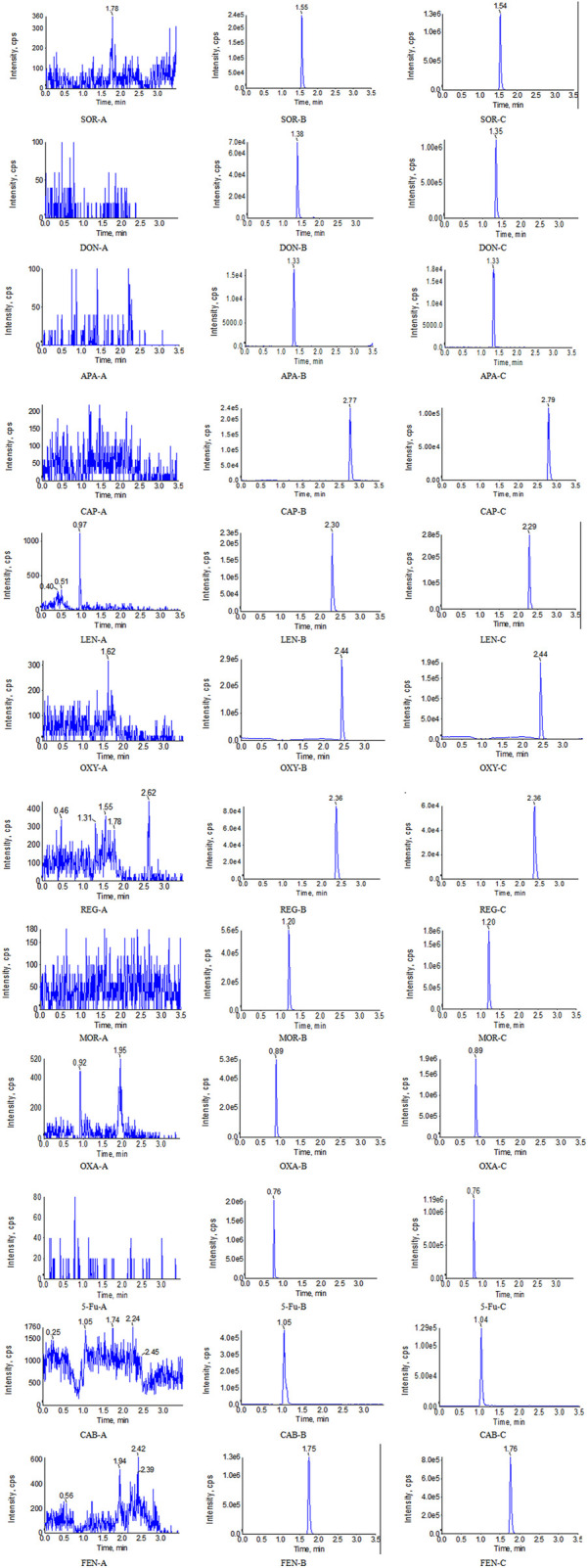
Representative UPLC-MS/MS chromatograms for SOR, DON, APA, CAP, LEN, OXY, REG, MOR, OXA, 5-Fu, CAB, and FEN in human plasma samples: **(A)** a blank plasma sample; **(B)** a blank plasma sample spiked with analyte, and **(C)** a clinical plasma sample.

The optimum chromatographic conditions were the basis of obtaining the ideal chromatographic peak, including an appropriate retention time, peak shape, low matrix effect and so on. In this study, gradient elution was performed at a flow rate of 0.4 mL/min. The specific gradient procedure was as follows: 0–0.4 min, 35% A (isocratic); 0.4–0.7 min, 35%–85% A (linear gradient); 0.7–3.0 min, 85% A (isocratic); 3.0–3.2 min, 85%–35% A (linear gradient); and 3.2–3.5 min, 35% A (isocratic). In addtion, we found that both acetonitrile and methanol as the organic phase could effectively elute all the analytes and ISs, with no obvious irregular chromatographic peaks. In comparison, the retention time of all compounds was within 3.5 min when acetonitrile was used as the organic phase. The proper column temperature can affect the viscosity of the mobile phase and thus the retention time. In this paper, the column temperature is set to 35°. Under these conditions, the retention time of CAB, SOR, APA, REG, LEN, DON, 5-Fu, OXA, CAP, MOR, OXY, and FEN was 1.04, 1.54, 1.33, 2.36, 2.30, 1.38, 0.76, 0.89, 2.77, 1.20, 2.44, and 1.75 respectively. The final chromatograms, ion pairs and main mass spectrometry parameters were generalized in Table 1 and Figure 2.

MS conditions were also repeatedly optimized in order to obtain strong and stable signal. Firstly, we found that the intensity of signal of positive ionization mode was more stable and stronger than negative ionization mode. Finally, the positive ion mode was employed. Secondly, different additives (formic acid, ammonium acetate) can affect the ionization of the analytes. Ammonium acetate had a significant inhibitory effect on the ionization of the analytes. In contrast, we found that 0.1% formic acid (v/v) contributed to the ionization of all the analytes and ISs.

Although plasma samples with high cleanliness and reproducibility can be obtained through mSPE technology, we should pay attention to the following points: 1) The polarity of drugs affects their retention behavior on magnetic particles, so appropriate mSPE particles should be selected according to the nature of drugs to avoid affecting the recovery. Generally speaking, compounds with general polarity can be adsorbed and separated based on the reverse phase mechanism (HLB). For weakly acidic and weakly alkaline compounds, mSPE particles with strong cationic materials and strong anionic materials can be selected as stationary phase respectively. For strongly acidic and strongly basic compounds, mSPE particles with weak cation exchange materials and weak anion exchange materials can be selected as stationary phase. Finally, the ionization state and retention behavior of the target compound were controlled by adjusting the solvent strength and pH value. 2) Plasma samples processed by mSPE technology cannot be concentrated, which is a challenge for the determination of drugs or metabolites with low concentrations. 3) After determining the type of mSPE particles, we should also systematically optimize the type and volume of eluent solution, and time of eluent. For example, we investigated different volumes of methanol and acetonitrile in this paper. The results showed that compared with methanol, higher recovery rate could be obtained when acetonitrile was selected as eluent solution. In the process of optimizing the volume of eluate, we should consider the recovery rate and the concentration of drug. Additionally, the third step was to complete the adsorption of mSPE particles to 7 ISs and 12 analytes. We found that excessive organic solvents will affect the adsorption of mSPE particles to ISs and analytes because of the elution effect of methanol. Finally, 15% methanol was used as diluting solvent to dilute the stock solution. On the one hand, it did not affect the adsorption of ISs and analytes; On the other hand, it can ensure the solubility of all ISs and analytes.

### 3.2 Method validation

#### 3.2.1 Specificity

Eight extracted blank samples were injected into the UPLC-MS/MS system to evaluate specificity. As shown in Figure 2, no other chromatographic peaks in blank human samples were shown, which indicated that there was no significant interference in the quantification of eight blank samples for analytes or ISs. Meanwhile, we found that 14 potential co-medications also did not interfere with the determination of the analytes and ISs.

#### 3.2.2 Linearity and LLOQ

All the standard curves showed satisfactory linearity over the concentration range of 10.0–10 000.0 *n*g/mL for SOR, CAB, REG, DON, APA, and LEN and 20.0–20 000.0 *n*g/mL for 5-Fu, OXA, CAP, MOR, OXY, and FEN. Typical standard curve equations were shown in Table 1. The LLOQ of SOR, CAB, REG, DON, APA, and LEN was 10.0 ng/mL, while the LLOQ of 5-Fu, OXA, CAP, MOR, OXY, and FEN was 20.0 ng/mL. There was no significant or unacceptable carryover effect for the blank samples in the ion channel of analytes and ISs after injection at the ULOQ.

#### 3.2.3 Precision and accuracy

As displayed in Table 2, the precision for SOR, CAB, REG, DON, APA, LEN, 5-Fu, OXA, CAP, MOR, OXY, and FEN ranged from 0.89% to 7.21%, while the accuracy of all the analytes ranged from −4.18% to 5.78%. Thus, the proposed method exhibited pretty good accuracy and precision.

**TABLE 2:** Methodology verification results of precision, accuracy, extraction recovery and matrix effect.

Drug	QC concentration (ng/mL)	Intra-day		Inter-day		Mean recovery (RSD%)	Mean matrix effect (RSD%)
		RSD%	RE%	RSD%	RE%		
	20.0	5.12	1.76	1.11	−2.22	98.9 (4.1)	96.0 (1.6)
5-Fu	40.0	0.99	−2.68	3.72	1.00	97.4 (3.5)	98.1 (3.0)
	400.0	2.74	3.55	2.72	2.48	93.2 (2.3)	99.7 (4.1)
	4,000.0	4.78	4.22	5.01	3.78	93.2(5.4)	95.3 (4.7)
	16,000.0	3.33	1.77	4.01	−1.08	102.7 (4.0)	104.4 (4.4)
	20.0	5.27	1.08	4.18	3.61	97.3 (2.8)	98.8 (3.6)
OXA	40.0	1.83	2.44	1.70	4.28	96.6 (3.5)	94.4 (3.1)
	400.0	3.42	4.67	4.34	5.78	99.2 (1.8)	96.0 (4.0)
	4,000.0	3.72	2.91	4.55	3.60	93.4 (4.9)	102.6 (4.2)
	16,000.0	2.70	2.99	3.78	4.22	97.4 (2.9)	103.2 (2.1)
	20.0	4.99	−2.61	1.94	−4.02	98.0 (3.5)	98.2 (4.0)
CAP	40.0	7.21	3.55	0.95	−3.64	97.4 (2.7)	93.8 (2.6)
	400.0	2.56	2.04	2.88	4.11	103.9 (1.7)	97.5 (3.5)
	4,000.0	5.20	2.79	4.81	−4.74	93.4 (2.9)	95.3 (4.2)
	16,000.0	3.55	2.93	1.79	5.01	100.3 (3.2)	98.8 (3.1)
	10.0	1.74	2.74	2.45	1.89	95.2 (2.2)	97.0 (4.7)
SOR	20.0	1.90	−0.93	1.24	4.66	96.9 (4.7)	99.3 (2.6)
	200.0	3.08	2.66	3.26	2.71	105.8 (3.5)	96.9 (1.4)
	2,000.0	4.14	3.57	2.69	3.02	96.4 (5.9)	95.5 (4.4)
	8,000.0	4.29	3.75	4.72	−2.22	101.1 (1.6)	97.7 (3.6)
	10.0	2.22	2.63	1.90	1.72	93.9 (2.9)	98.1 (3.2)
CAB	20.0	1.99	4.02	2.33	3.28	94.8 (4.3)	96.5 (2.9)
	200.0	2.05	2.33	2.68	2.60	99.1 (2.8)	105.9 (3.1)
	2,000.0	3.00	3.28	4.17	−3.45	94.2 (5.1)	97.7 (2.9)
	8,000.0	5.70	−4.18	3.59	−1.55	98.5 (3.1)	99.7 (2.9)
	10.0	3.20	2.69	1.27	−1.92	98.7 (3.2)	99.5 (2.6)
DON	20.0	3.93	3.58	1.80	2.64	97.8 (3.1)	99.0 (3.1)
	200.0	2.00	3.43	5.22	1.95	101.0 (2.7)	99.9 (1.5)
	2,000.0	3.33	−2.94	3.20	1.74	97.2 (4.0)	94.8 (3.7)
	8,000.0	1.56	−2.57	3.12	5.62	99.0 (2.9)	101.8 (3.6)
	10.0	2.78	4.33	2.16	3.33	100.7 (2.6)	99.7 (1.8)
REG	20.0	3.53	1.94	3.50	1.73	95.2 (4.5)	97.3 (3.3)
	200.0	2.85	2.65	3.27	2.55	97.9 (3.7)	106.2 (2.4)
	2,000.0	2.07	−3.21	4.56	3.88	102.4 (5.1)	96.2 (6.6)
	8,000.0	0.89	2.87	2.76	5.00	96.4 (2.9)	96.0 (2.5)
	10.0	1.05	−1.99	4.88	3.45	94.5 (0.9)	99.6 (3.3)
LEN	20.0	1.88	4.24	4.22	4.28	98.3 (1.6)	96.4 (2.9)
	200.0	3.72	5.32	3.60	−2.54	99.2 (4.6)	97.9 (5.2)
	2,000.0	3.76	2.44	4.90	−3.17	95.2 (6.9)	96.1 (8.4)
	8,000.0	5.11	−3.79	1.79	3.40	97.4 (2.0)	98.0 (2.4)
	10.0	3.17	1.66	2.75	4.32	96.9 (2.5)	99.3 (4.2)
APA	20.0	2.08	3.26	4.33	2.71	98.8 (3.7)	97.2 (2.8)
	200.0	1.80	−1.83	1.89	2.22	101.1 (1.7)	97.8 (3.0)
	2,000.0	3.64	2.07	4.58	4.19	93.2 (5.3)	99.3 (3.0)
	8,000.0	1.52	2.44	4.37	−3.67	100.0 (3.3)	94.3 (1.9)
	20.0	6.63	2.64	2.66	2.00	99.9 (3.1)	95.5 (1.3)
MOR	40.0	3.62	3.15	3.21	4.62	94.0 (4.0)	97.0 (2.5)
	400.0	1.85	1.83	5.34	1.78	97.8 (2.7)	99.4 (3.9)
	4,000.0	3.79	4.88	4.20	−2.99	94.3 (5.0)	95.5 (2.1)
	16,000.0	2.49	1.42	2.33	2.59	100.7 (3.6)	99.1 (2.2)
	20.0	3.61	3.33	2.78	−3.62	98.0 (4.6)	96.0 (3.6)
	40.0	2.37	1.08	2.41	3.17	100.3 (2.2)	98.7 (4.4)
OXY	400.0	4.44	1.71	3.04	2.66	93.1 (3.1)	102.3 (2.3)
	4,000.0	3.89	2.71	4.27	0.88	97.5 (4.1)	95.2 (5.2)
	16,000.0	4.19	2.94	2.59	1.72	97.2 (2.0)	99.5 (2.7)
	20.0	2.38	4.21	3.75	−0.94	97.4 (1.5)	98.3 (3.9)
FEN	40.0	2.95	−2.74	3.86	2.16	95.0 (1.9)	96.3 (3.2)
	400.0	1.42	2.66	2.11	4.17	93.6 (3.3)	93.7 (0.7)
	4,000.0	3.33	−2.87	4.27	−2.06	98.4 (4.4)	93.0 (4.6)
	16,000.0	2.04	3.81	3.48	3.25	96.8 (2.6)	94.9 (1.0)

#### 3.2.4 Recovery and matrix effect

The matrix effect for all the analytes and ISs at HQC, MQC, LQC, and LLOQ level ranged from 93.1% to 105.8% and 94.75%–99.07% respectively. Meanwhile, the extraction recovery for all the analytes and ISs ranged from 93.7% to 106.9% and 96.8%–100.8% respectively. In addition, the recovery and matrix effect of all analytes were < 6.9% and 8.4%, respectively. It can be seen from Table 2 that the matrix effect was negligible in this study.

#### 3.2.5 Stability

Table 3 summarizes the stability results for SOR, DON, REG, LEN, CAB, APA, 5-Fu, OXA, CAP, MOR, OXY, and FEN in human plasma after storage. SOR, DON, REG, LEN, CAB, APA, 5-Fu, OXA, CAP, MOR, OXY, and FEN were stable in human plasma. Importantly, although the stability was acceptable, the concentration of OXA decreased significantly after three freeze-thaw cycles, about 9.47%. In order to further investigate the effect of freeze-thaw cycles on the stability of OXA, we also determined the concentration of OXA after one freeze-thaw cycle and two freeze-thaw cycles. The results showed that the concentration of OXA decreased by (2.43%, 1.89%, 2.20%, and 1.79%) and (4.17%, 3.56%, 4.88%, and 3.41%) after one freeze-thaw cycle and two freeze-thaw cycles at four QC level, respectively. Therefore, multiple freeze-thaw cycles may affect the stability of OXA.

**TABLE 3:** Stability and dilution integrity of analytes in plasma under various storage conditions (data are mean RE %, *n* = 4).

Drug	QC concentration (μg/mL)	Room temperature	−70°C for 30 days	Freeze-thaw cycles	Autosampler stability	Dilution integrity	
						5-Fold	50-Fold
	40.0	3.28	2.69	2.57	−2.45	____	____
5-Fu	400.0	1.67	1.79	4.00	3.61	____	____
	4,000.0	4.66	−3.79	3.68	1.72	____	____
	16,000.0	1.49	0.77	3.69	−2.99	0.74	1.79
	40.0	−2.55	−2.95	−9.47	3.74	____	____
OXA	400.0	−1.78	2.77	−8.19	−2.89	____	____
	4,000.0	4.61	2.93	−9.01	−3.71	____	____
	16,000.0	2.00	−3.46	−7.60	−2.52	2.49	−3.18
	40.0	1.89	2.10	1.82	2.71	____	____
CAP	400.0	0.99	1.80	2.35	1.52	____	____
	4,000.0	3.21	2.79	4.44	3.58	____	____
	16,000.0	3.43	2.88	0.77	4.23	2.81	1.73
	20.0	−2.73	−1.79	2.63	2.40	____	____
SOR	200.0	3.10	3.39	−1.08	2.88	____	____
	2,000.0	−3.94	2.74	−2.98	3.01	____	____
	8,000.0	2.71	2.63	2.84	−4.10	1.55	2.41
	20.0	−4.37	3.29	−2.57	3.49	____	____
CAB	200.0	4.62	0.51	2.77	2.41	____	____
	2,000.0	2.69	4.89	−2.02	2.42	____	____
	8,000.0	2.94	3.79	−3.28	2.72	−1.76	−2.84
	20.0	−2.88	−0.94	1.32	−3.90	____	____
DON	200.0	3.00	−4.54	2.53	3.99	____	____
	2,000.0	4.88	3.71	3.05	2.94	____	____
	8,000.0	−2.47	3.21	−0.78	2.90	2.03	4.75
	20.0	3.42	−3.01	4.29	1.49	____	____
REG	200.0	−5.75	−3.11	1.73	3.66	____	____
	2,000.0	−4.08	3.94	−0.83	2.56	____	____
	8,000.0	2.49	3.07	−2.55	1.73	3.82	−0.77
	20.0	1.11	2.50	3.57	2.99	____	____
LEN	200.0	−0.76	3.92	0.74	−4.05	____	____
	2,000.0	−3.79	−2.22	−3.08	1.67	____	____
	8,000.0	3.58	−2.64	−4.27	0.86	2.57	1.00
	20.0	3.75	2.60	2.98	3.47	____	____
APA	200.0	2.68	3.82	−4.82	2.58	____	____
	2,000.0	4.58	1.08	3.52	2.35	____	____
	8,000.0	−1.04	1.04	−2.73	1.09	2.48	3.33
	40.0	2.69	2.66	2.01	−0.71	____	____
MOR	400.0	−3.01	−2.59	1.39	2.85	____	____
	4,000.0	1.64	2.72	3.01	1.39	____	____
	16,000.0	2.35	3.27	−1.23	1.89	2.40	1.58
	40.0	−2.30	1.57	3.88	2.73	____	____
FEN	400.0	1.44	2.99	1.60	3.06	____	____
	4,000.0	3.72	−2.65	−3.88	1.09	____	____
	16,000.0	2.64	−0.65	1.06	−2.54	−3.21	−1.99
	40.0	1.45	4.56	−2.77	3.05	____	____
OXY	400.0	−1.95	2.93	−1.40	2.11	____	____
	4,000.0	−3.62	−4.17	2.97	1.09	____	____
	16,000.0	2.63	1.77	1.03	1.29	2.60	2.74

#### 3.2.6 Dilution integrity

The accuracy of dilution integrity ranged from −3.21% to 4.75%, which were within the criterion (±15%). The final results of dilution integrity were shown in Table 3. It proved that the method to measure all the analytes by diluting with blank plasma was accurate, reliable and reproducible.

### 3.3 Application

By using the validated UPLC–MS/MS method, we successfully determined 45 5-Fu, 70 CAP, 115 OXA, 95 SOR, 32 DON, 30 REG, 118 LEN, 20 CAB, 41 APA, 49 MOR, 52 OXY, and 37 FEN. The method could accurately detect and measure the plasma drug concentrations of SOR, DON, REG, LEN, CAB, APA, 5-Fu, OXA, CAP, MOR, OXY, and FEN (Table 4). The results showed that the plasma drug concentrations among patients showed great intra- and inter-individual variability under similar dosing schedule and blood collection time, especially for 5-Fu, CAP, OXA, SOR, REG, and FEN. Meanwhile, the efficacy and toxic side effects, which were highly correlated with plasma drug concentrations, also showed large individual differences. The following factors may be related to the fluctuation of plasma drug concentration, such as 1) Genetic polymorphisms of metabolic enzymes can significantly affect the concentration of certain drugs. 2) Drug–drug interactions associated with metabolic enzymes can significantly alter drug concentrations. 3) The enterohepatic recycling effect can promote the reabsorption of drugs in the duodenum, thus prolonging the duration of the drug in the body and causing the appearance of double peaks on the concentration-time curve, ultimately affecting plasma concentration and bioavailability. 4) The status of HCC can affect the activity of drug-metabolizing enzymes in the liver, thereby altering drug concentrations. With the occurrence and development of HCC, the expression level of the CYP enzyme protein in tumor tissue would be reduced, but the reduction in the activity of various enzymes varies significantly, resulting in gene polymorphisms of metabolic enzymes and affecting the metabolism of substrates (Gao et al., 2016). 5) Other drugs commonly used in HCC patients, such as hepatoprotective, choleretic, and antiviral drugs, may affect the concentration of HCC treatment drugs. In addition, fluctuations in plasma drug concentrations due to interactions between drugs with high plasma protein-binding rates should also be considered. The plasma protein binding rate of SOR, LEN, RGE, and FEN reached 99.5%, 99.0%, 99.5%, and 85.0%, respectively, while the protein binding rate of the choleretic drug ursodeoxycholic acid was 96%–99%. Therefore, the potential of drug replacement to change plasma drug concentrations requires particular attention.

**TABLE 4:** Determination results of the plasma concentration of SOR, DON, REG, LEN, CAB, APA, 5-Fu, OXA, CAP, MOR, OXY, and FEN in patients.

Drug	Quantity of TDM	Dose regiment	Plasma concentration
OXA + 5-Fu	45	5-Fu: 400 mg/m^2^, iv + 600 mg/m^2^, ivgtt	5-Fu: 1.75 μg/mL∼31.8 μg/mL
		OXA: 85 mg/m^2^/each time, ivgtt	OXA: 1.24 μg/mL∼13.83 μg/mL
OXA + CAP	70	CAP: 1,000 mg/m^2^/each time, po, bid, continuously	CAP: 1.22 μg/mL∼25.3 μg/mL
		OXA: 130 mg/m^2^/each time, ivgtt	OXA: 1.39 μg/mL∼13.41 μg/mL
SOR	95	Po, 400 mg/each time, bid, continuously	0.56 μg/mL∼6.22 μg/mL
DON	32	Po, 200 mg/each time, bid, continuously	0.74 μg/mL∼6.48 μg/mL
REG	30	Po, 160 mg, qd, continuously	0.37 μg/mL∼4.61 μg/mL
LEN	118	Po, 12 mg, qd, continuously	24.5 ng/mL∼169.3 ng/mL
CAB	20	Po, 60 mg, qd, continuously	0.54 μg/mL∼3.75 μg/mL
APA	41	Po, 750 mg, qd, continuously	0.37 μg/mL∼2.14 μg/mL
MOR	49	Po, 30 mg/each time, bid, continuously	15.7 ng/mL∼49.7 ng/mL
OXY	52	Po, 40 mg/each time, bid, continuously	3.8 ng/mL∼28.5 ng/mL
FEN	37	Transdermal patch, 8.4 mg/patch, q72 h	1.1 ng/mL∼12.4 ng/mL

## 4 Conclusion

In this paper, we developed and validated a sensitive, rapid, reliable, and accurate UPLC–MS/MS method for the simultaneous quantification of 5-Fu, OXA, CAP, SOR, REG, LEN, APA, DON, CAB, MOR, OXY, and FEN in human plasma. This method was successfully applied in the clinical TDM of three chemotherapy drugs, six targeted drugs, and three analgesics commonly used in the treatment of HCC. We also discussed the potential factors affecting plasma drug concentrations, with specific reference to drug interactions, metabolic enzyme polymorphisms, HCC status, and concomitant medications. Our study provides an empirical basis for adoption of the proposed UPLC–MS/MS method in clinical TDM and pharmacokinetic studies.

## Data Availability

The original contributions presented in the study are included in the article/supplementary material, further inquiries can be directed to the corresponding author.
